# Oligomerization of Sticholysins from Förster Resonance Energy Transfer

**DOI:** 10.1021/acs.biochem.0c00840

**Published:** 2021-01-14

**Authors:** Juan Palacios-Ortega, Esperanza Rivera-de-Torre, Sara García-Linares, José G. Gavilanes, Álvaro Martínez-del-Pozo, J. Peter Slotte

**Affiliations:** † Departamento de Bioquímica y Biología Molecular, Universidad Complutense, 28040 Madrid, Spain; ‡ Biochemistry, Faculty of Science and Engineering, Åbo Akademi University, 20520 Turku, Finland

## Abstract

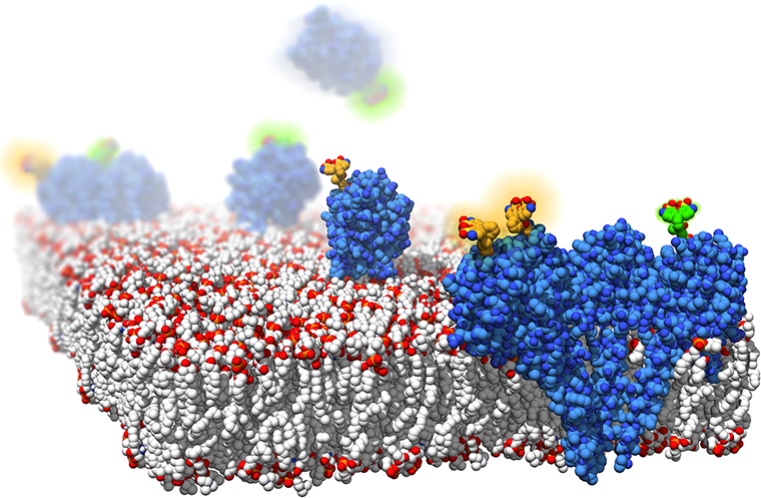

Sticholysins are pore-forming toxins produced by sea anemones that are members of the actinoporin family. They exert their activity by forming pores on membranes, provided they have sphingomyelin. To assemble into pores, specific recognition, binding, and oligomerization are required. While recognition and binding have been extensively studied, delving into the oligomerization process and the stoichiometry of the pores has been more difficult. Here, we present evidence that these toxins are capable of oligomerizing in solution and suggesting that the interaction of sticholysin II (StnII) with its isoform sticholysin I (StnI) is stronger than that of StnI with itself. We also show that the stoichiometry of the final, thermodynamically stable StnI pores is, at least, heptameric. Furthermore, our results indicate that this association maintains its oligomerization number when StnII is included, indicating that the stoichiometry of StnII is also of that order, and not tetrameric, as previously thought. These results are compatible with the stoichiometry observed for the crystallized pore of FraC, another very similar actinoporin produced by a different sea anemone species. Our results also indicate that the stoichiometry of actinoporin pores in equilibrium is conserved regardless of the particular composition of a given pore ensemble, which we have shown for mixed sticholysin pores.

The production of venom containing very similar toxins is a common feature of sea anemones around the globe.^1−8^ Sticholysins are similar toxic proteins produced by the Caribbean Sea anemone *Stichodactyla helianthus*.^9,10^ They belong to a family of cytolytic proteins known as actinoporins. They are pore-forming toxins and, as such, exert their activity by creating pores in selected target membranes.^11−13^ These membranes feature sphingomyelin (SM), a lipid that is specifically recognized by these proteins and whose absence in the membranes of sea anemones provides the basis for avoiding self-toxicity.^9,10,14−19^

Thanks to years of research, many details of the functionality of these proteins have been elucidated. The knowledge acquired includes the role of specific residues and the influence of the lipid composition of the membrane. SM selectivity, the role of tryptophan residues, the enhancing effect of cholesterol (Chol) on activity, and the crucial intervention of the N-terminal α-helix in the process of pore formation are some of the aspects that have been elucidated.^9,10,14−16,20−26^ Studies have traditionally been performed using one (or several) of the following proteins: sticholysins I and II (StnI, UniProtKB P81662; and StnII, UniProtKB P07845), equinatoxin II (EqtII), and/or fragaceatoxin C (FraC). The soluble, monomeric structures of these four proteins have been revealed in atomic detail.^7,27−30^ All of them display a common fold, which consists of a β-sandwich flanked by two α-helices (Figure 1). The α-helix located at the N-terminus is responsible for membrane penetration.^22−26^

**Figure 1:**
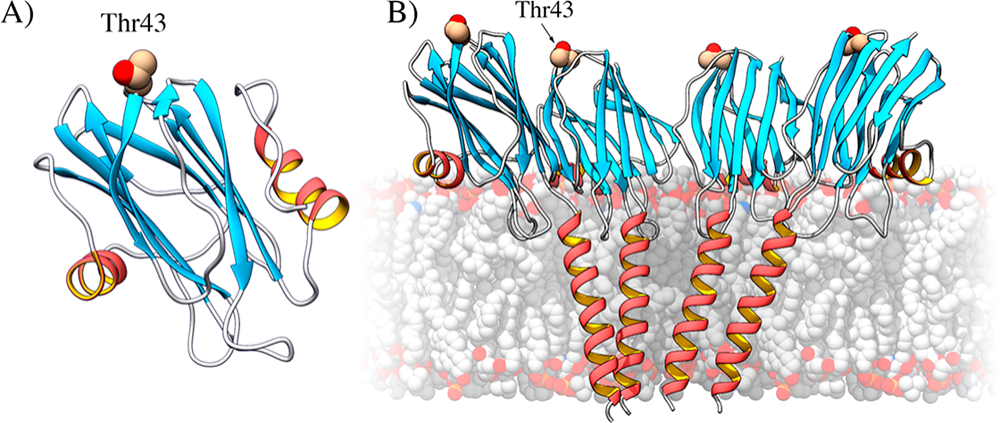
(A) Position of the mutated residue, Thr43, in the structure of StnI (Protein Data Bank entry 2KS4). Its replacement by a Cys residue would not affect the membrane-binding region (α-helix below) or the protein–protein interface (side of the protein in the front). (B) Mutated residue in the context of an oligomer, showing that it is unlikely to interfere with oligomerization (see Figure S2 for further details). The figure was made by fitting the structure of StnI to that of the octameric pore of FraC (Protein Data Bank entry 4TSY). Made with UCSF Chimera.^31^

The aforementioned soluble structures were obtained using X-ray crystallography and/or nuclear magnetic resonance. However, the structure of the pore complex into which these toxins assemble in SM-containing membranes, revealing all of the structural changes and the stoichiometry of these complexes, has not been easy to resolve. To penetrate the membrane and form a pore, the structure of an actinoporin has to undergo conformational changes. These changes include oligomerization, the concomitant monomer–monomer contacts, and the deployment and extension of the N-terminal α-helix, the most significant one at the monomer level. All of these are structural modifications that take place exclusively in the presence of suitable membranes.

Therefore, the presence of lipids appears to be necessary if the complexes formed by actinoporins are to be understood. Accordingly, early attempts to understand these complexes used StnII crystallized on egg phosphatidylcholine (egg-PC) and dioleoylphosphatidylcholine (DOPC) monolayers, with the crystallization process appearing to force membrane binding despite the absence of SM.^28^ These experiments yielded three-dimensional maps at 18 Å resolution that were used to fit the previously available structure of the soluble monomers. In this case, it appeared that the stoichiometry of StnII pores was tetrameric. Years later, a new approach was devised. It overcame the limitation imposed by lipids for X-ray crystallography by removing or replacing them with detergents in a way that, in principle, conserved the lipid–protein and protein–protein interactions that stabilize the membrane-bound conformation of actinoporins.^32,33^ These experiments were performed using FraC. Several structures were obtained, including that of a nonameric prepore ensemble and, most importantly, an octameric pore, at a resolution of 3.2 Å.^33^

Tetrameric, heptameric, and octameric ensembles, represented by the toroidal pore, the conical pore, and the hybrid pore models, are currently the most accepted candidates for the pore structures in the field of actinoporins. There are, however, many clear indications about the simultaneity of several other stoichiometries during the process leading to the formation of the final pore structures in equilibrium. Nevertheless, some questions remain unanswered or, at least, poorly understood. For example, is the stoichiometry of the pores of all actinoporins the same? Are multiple stoichiometries possible under the same conditions? Could it be that detergent treatments favor the formation of specific complexes? Do the final pore structures constitute a thermodynamically stable assembly? Do different toxin isoforms produced by the same sea anemone species assemble into the same structure and yield heteropores?

To answer these questions, we conceived a new approach, taking advantage of the fact that the wild-type (WT) variants of most actinoporins lack cysteine (Cys) residues in their sequences. Building on the accumulated functional and structural knowledge on actinoporins, and particularly sticholysins, we created a single-cysteine mutant, StnI-T43C, that enabled specific labeling with a high-quantum yield fluorescent probe while keeping functional disturbance to a minimum. Using this mutant, we have been able to study the behavior of these proteins in solution and on membranes and, using a FRET (Förster resonance energy transfer) approach, to elucidate the stoichiometry of StnI and StnI–StnII pores.

## Materials and Methods

### Materials

1,2-Dioleoyl-*sn*-glycero-3-phosphocholine (DOPC), 1-palmitoyl-2-oleoyl-*sn*-glycero-3-phosphocholine (POPC), *N*-palmitoyl-d-*erythro*-sphingosylphosphorylcholine (PSM), and egg sphingomyelin (eSM) were obtained from Avanti Polar Lipids (Alabaster, AL). Maleimide-modified ATTO-488 and ATTO-542 were obtained from Atto-Tec GmbH (Siegen, Germany). All sticholysin variants were produced in *Escherichia coli*, strain RB791, and purified to homogeneity as described in refs (21) and (34). Briefly, purification was performed using ion-exchange chromatography (carboxymethyl-cellulose, CM-52) eluted using a linear gradient of NaCl (0 to 0.3 M) followed by size-exclusion chromatography (Biogel P2). Homogeneity of the preparation was evaluated by means of sodium dodecyl sulfate–polyacrylamide gel electrophoresis and amino acid analysis.

### Methods

#### Protein Preparation

The mutant StnI-T43C was produced using site-directed mutagenesis. Its design takes advantage of the lack of Cys residues in sticholysins, enabling them to be specifically labeled at a convenient position, safe from the perspective of functionality, where the label is not expected to interfere with either protein–protein interactions or membrane recognition and binding (Figure 1).

The new mutant was characterized structurally and functionally using circular dichroism, and hemolytic assays as previously described.^35^ Its hemolytic activity was essentially the same as that of the WT StnI variant, indicating that the mutation did not affect any functionally important region of the protein (Figure S1), as expected from the design (Figure 1 and Figure S2). For these assays, the mutant was previously incubated with 0.5 mM TCEP to ensure that all potential disulfide bonds were reduced.

StnI-T43C was specifically labeled at the introduced -SH group using maleimide-modified ATTO probes (5–8-fold molar excess) overnight after a previous 2 day incubation with TCEP [tris(2-carboxyethyl)phosphine; 50:1 molar excess] in phosphate buffer (140 mM NaCl and 10 mM phosphate) at pH 7.4. The excess of free label was effectively removed using Pierce Dye Removal Columns (Thermo Fisher Scientific) following the manufacturer’s specifications. The yield of the labeling procedures ranged between 17% and 25%, depending on the batch. The efficiency of labeling was evaluated by means of the absorption spectra of the sample, using the following extinction coefficients: ε = 49450 M^–1^ cm^–1^ for StnI-T43C at 280 nm, ε = 9.0 × 10^4^ M^–1^ cm^–1^ at 500 nm for ATTO-488, and ε = 1.2 × 10^5^ M^–1^ cm^–1^ at 542 nm for ATTO-542. Corrections for label contributions at 280 nm were made using the corresponding correction factors provided by the manufacturer, which are 0.09 and 0.08 for ATTO-488 and ATTO-542, respectively (see Figure S3 for the complete absorption spectra). These correction factors represent the absorbance of the label at 280 nm relative to their respective maximum absorbances.

#### Vesicle Preparation

Lipid vesicles were prepared by mixing selected methanol (hexane for Chol) solutions of lipids in the desired lipid molar proportion. The organic solvent was evaporated under a nitrogen flow at 40 °C. Dried lipid films were then hydrated at 65 °C in a water bath for at least 30 min. Suspended lipid vesicles were then extruded at hydration temperature through 200 nm diameter polycarbonate filters.

#### Time-Resolved Fluorescence Anisotropy

Time-resolved fluorescence measurements were performed using a FluoTime100 spectrofluorimeter equipped with a PicoHarp300E time-correlated single photon-counting module (PicoQuant GmbH, Berlin, Germany) and polarizers. A 457 ± 15 nm pulsed diode laser was used for excitation. Emission was collected through a long-pass filter (>480 nm). When required, neutral density filters were used to attenuate the excitation intensity. The instrument response function (IRF) was acquired using light scattered by buffer in the absence of colored filters up to 10000 counts in the peak channel. Each sample decay was recorded up to ∼20000 counts in the peak channel. To avoid inner filter effects, the concentration of the fluorophores was such that the optical density at the excitation wavelength was OD_*l*/2_ < 0.05. Experiments were performed under constant stirring. The temperature was controlled by a Peltier element.

Anisotropy decays were analyzed using FluoFit Pro software from PicoQuant. The *G* factor was recorded for each sample and was always between 0.98 and 1.02. The fluorescence anisotropy decay curves [*r*(*t*)] were fit using a model consisting of the sum of discrete exponential terms:^36^
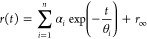
1where α_*i*_ and θ_*i*_ are the normalized amplitude and the rotational correlation time of the *i*th component of the anisotropy decay, respectively. The symbol *r*_∞_ is the limiting anisotropy, which is related to the restrictions in the process of depolarization. The number of exponential terms was always the smallest required to obtain a satisfactory fit, as judged from the value of the reduced χ^2^, the distribution of the residuals, and the trace of the autocorrelation plot.

Rotational correlation times were used to estimate molecular diameters, according to
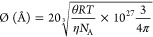
2which is derived from the Perrin equation,^36^ modified to yield the molecular diameter directly in angstroms, where θ is the correlation time of the rotating unit in seconds, *R* is the ideal gas constant (8.314 m^3^ Pa mol^–1^ K^–1^), *T* is the temperature in kelvin, η is the viscosity of the solvent (0.94 × 10^–3^ Pa s), and *N*_A_ is Avogadro’s number. The numerical constants appearing in the equation transform the radius into the diameter while also transforming the output units. When using this equation, an assumption is made that the overall shape of the rotating unit is that of a sphere. This can be expected for molecules that display a single correlation time.^36^

#### Calculation of the Förster Distance

The Förster distance (*R*_0_) of the donor–acceptor pair used was calculated as
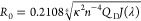
3which yields *R*_0_ directly in angstroms. In the expression, κ^2^ is the orientation factor, set to ^2^/_3_, which represents the dynamic isotropic limit (see Figure S4), *n* is the refractive index of the medium, set to 1.33 (water), *Q*_D_ is the quantum yield of the donor (0.8, according to the supplier), and *J*(λ) is the overlap integral between the emission spectra of the donor and the absorption spectra of the acceptor. The overlap integral is calculated as
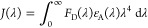
4where *F*_D_(λ) is the emission spectra of the donor with its area normalized to 1 and ε_A_(λ) is the acceptor spectra in M^–1^ cm^–1^. The calculated *R*_0_ for the ATTO-488/ATTO-542 FRET pair was 63.8 Å. The *R*_0_ for the ATTO-488 self-transfer was 49.3 Å.

#### Steady-State Fluorescence Spectroscopy

Steady-state fluorescence measurements were performed on a PTI QuantaMaster spectrofluorimeter (Photon Technology International, Lawrenceville, NJ). Sample excitation was set at 500 nm. Emission was recorded between 507 and 675 nm to encompass the spectra of both fluorophores. To avoid inner filter effects, the concentration of the fluorophores was such that the optical density at the excitation wavelength was OD_*l*/2_ < 0.05. Experiments were performed under constant stirring. The temperature was controlled by a Peltier element.

#### Stoichiometry of Oligomers from FRET

A model was constructed to calculate the expected FRET efficiencies for each possible stoichiometry in a way that was dependent on the fraction of acceptor-labeled toxin in the sample, while the fraction of donor was kept constant. Single-point mutants allowed the assumption that the fluorophores were distributed as the vertices of regular polygons (see Figure S5 for further details about this and the method). The distance from the fluorophores to the center of the polygon is expressed as
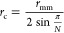
5where *r*_c_ is the radius of the polygon, *r*_mm_ is the distance between fluorophores on adjacent subunits, and *N* is the number of subunits per pore.

The value for *r*_mm_ was 29.0 Å, as measured for a sticholysin subunit (calculated from the correlation times). However, *r*_c_ cannot be used directly. Instead, the offset of the fluorophore relative to the center of the protein has to be taken into account. The distance to the center of the polygon to the fluorophore is then
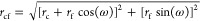
6where *r*_f_ is the distance from the mass center of the protein to the labeled position as observed from above (distance on the *z*-axis is not required) and ω is the angle between the line that joins them and the prolongation of the line that unites the center of the monomer and the center of the oligomer. The values of *r*_f_ and ω were measured as 10 Å and 66°, respectively, based on the position of residue T43 on the three-dimensional structure of StnI [Protein Data Bank (PDB) entry 2KS4].

Once *r*_cf_ is known, the distance between any two subunits can be calculated as
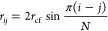
7where *i* and *j* are the indices of the subunits of interest. Usually, assuming *j* = 1, as will be done from here onward (omitting subscript *j*), eq 7 yields the distance between the selected subunit (number 1) and the *i*th subunit. Distance values were then used to calculate the corresponding rates of energy transfer, according to
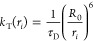
8where τ_D_ is the lifetime of the donor, which in our case is 4.1 ns.

To ensure the greatest precision, all possible arrangements of all possible combinations of unlabeled and donor- and acceptor-labeled subunits were calculated for each of the stoichiometries considered, assuming that monomer associations were random and unbiased by the labeling. This was achieved with a homemade Python program. The arrangements of subunits were calculated taking into account possible redundancies due to rotational symmetry. In all cases, it must be maintained that *N*_D_ + *N*_A_ + *N*_U_ = *N* [i.e., the sum of unlabeled (*N*_U_), donor-labeled (*N*_D_), and acceptor-labeled (*N*_A_) subunits must equal the total number of subunits].

For each of the possible arrangements, the expected FRET efficiency was calculated using the additive property of rates,^36^ as
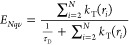
9where *E*_*Nq*ν_ indicates that it is the FRET efficiency for a stoichiometry of *N* subunits (for example, *N* = 8), a combination *q* of unlabeled and donor- and acceptor-labeled subunits (for example, *N*_D_ = 1, *N*_A_ = 3, and *N*_U_ = 4), ordered in a particular arrangement ν. For each arrangement, the observed FRET efficiency will depend on how the subunits are ordered within the oligomer. We assumed that monomer associations were random and unaffected by the labeling; hence, the observed FRET efficiency for each combination *q* will be the average of the *E* values for all of the possible arrangements, because we are assuming that all arrangements are equally possible within each combination.

The observed FRET efficiency would then depend on the fraction of donor- and acceptor-labeled toxins in the sample (*f*_A_ and *f*_D_, respectively). These fractions control which combinations are more likely to occur. The respective fractions of labeled and unlabeled subunits must equal one (i.e., *f*_A_ + *f*_D_ + *f*_U_ = 1). The probability of each combination, including all of its possible arrangements, is calculated as
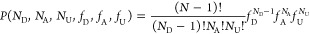
10which is a version of the trinomial distribution adapted to rule out redundancies due to rotational symmetries. The sum of all probabilities calculated in this manner must equal 1. This probability is the weighting factor for the energy transfer efficiencies of each combination. The final result yields the expected FRET efficiency for a given stoichiometry and fraction of labeling.

FRET efficiencies were measured in a large lipid:protein (L:P) molar ratio to avoid energy transfer between different pore complexes. The estimation of the right L:P ratio was made as , where *r*_av_ is the average distance between oligomers and σ is the surface density of the complexes.^37^ Thus, if the stoichiometry was 8, the average separation between complexes would be ∼3*R*_0_. Under these conditions, if the stoichiometry was equal to 4, the average separation would still be ∼2*R*_0_, at which the energy transfer efficiency is <2%. Larger L:P ratios could not be used due to experimental limitations, namely, light scattering by large unilamellar vesicles (LUVs), the availability of materials, and instrument sensitivity. Comparison of the experimental data with the model using root-mean-square deviations (RMSDs) allowed us to estimate the stoichiometry of the pores of sticholysins. For the calculation of the RMSDs, the error in each value was used to calculate weighting factors, so that the most accurate values contributed to the final RMSD.

Final protein mixtures were made by combination of aliquots from WT (StnI and StnII, as indicated) and labeled StnI (with donor or acceptor) stocks. The amounts of donor- and acceptor-labeled mutants were calculated according to the corresponding stock concentration and degree of labeling. The same procedure was used when StnII was included. WT StnI was added last, if needed, to adjust the final overall fraction of labeling and total protein concentration to the desired values. Throughout the study, StnI has been considered to be equal, structurally and in terms of activity, to the unlabeled StnI-T43C mutant, as evidenced by their practically indistinguishable CD spectra (both near and far UV) and hemolytic activity (Figure S1).

All fluorescence experiments were performed in PBS (10 mM phosphate and 140 mM NaCl) at pH 7.4.

## Results

### Motions of Sticholysins in Solution and on Membranes

**Figure 2:**
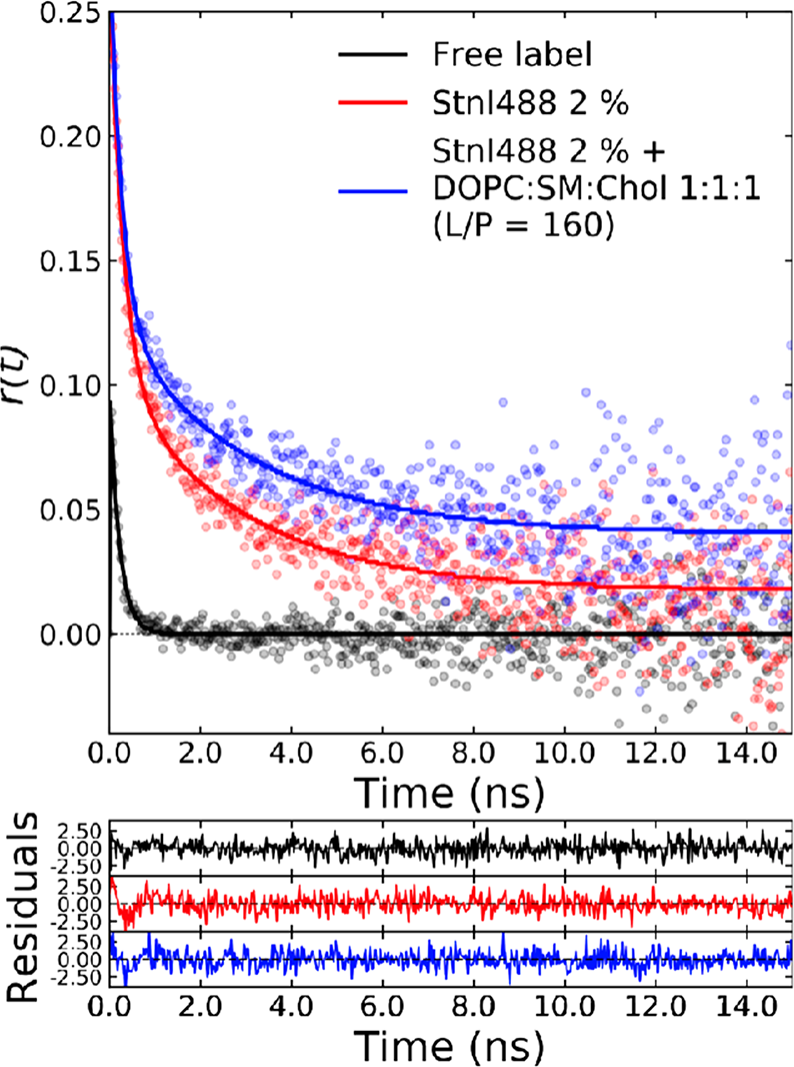
Time-dependent anisotropy decays of StnI labeled with ATTO-488 free in solution (degree of labeling of 2%, red) or in the presence of DOPC/eSM/Chol (1:1:1) membranes (L:P molar ratio of 160, blue). The decay of the free ATTO-488 label was also recorded (black). Traces of residuals for each of the fits are shown below. The order of the graphs is the same as that of the legend. The decay recorded using a 4:1 POPC:PSM ratio has been omitted for the sake of clarity.

StnI-T43C was labeled with ATTO-488. The time-resolved anisotropy decays of the toxin and the free label were recorded for the following reasons: (1) as a way to further ascertain that the labeling process had succeeded, (2) to observe if the label was able to interact with the LUVs by itself, and (3) with the intention of measuring the average hydrodynamic size of the StnI monomers. For these measurements, solutions in which only 2% of the total sticholysin was labeled were used (i.e., WT StnI was added to the 17–25% labeled mutant preparation to decrease the final overall degree of labeling to 2%) (Figure 2). Under these conditions, only a single, very short correlation time (0.2 ns) could be resolved for the free label. StnI displayed two correlation times. The faster one was ∼0.3 ns, very close to that of the free label, accounting for segmental motions of the fluorophore. The slower one was ∼2.95 ns and corresponded to the rotational motions of the proteins within the solvent (Table 1). This last correlation time could be used to estimate the molecular diameters of the different sticholysin species presumably present in the solution studied. The value obtained, 29.0 ± 1.3 Å, agrees with the molecular structures available, as demonstrated when placing a sphere at the mass centers of those structures. The time-dependent anisotropy did not decay to zero, displaying a limiting anisotropy of ∼0.014.

**Table 1:** Parameters of the Anisotropy Decays of ATTO-488, StnI (degree of labeling of 2%), in the Absence and Presence of Lipids (1:1:1 DOPC:eSM:Chol with an L:P molar ratio of 160; 4:1 POPC:PSM with an L:P molar ratio of 160)^a^

	*r*_1_	θ_1_ (ns)	*r*_2_	θ_2_ (ns)	*r*_*∞*_
ATTO-488	0.125 ± 0.005	0.228 ± 0.012	–	–	0.000 ± 0.0004
ATTO-488 with DOPC/eSM/Chol	0.135 ± 0.006	0.232 ± 0.012	–	–	0.001 ± 0.0004
StnI	0.200 ± 0.008	0.299 ± 0.030	0.089 ± 0.006	2.851 ± 0.530	0.014 ± 0.002
StnI with DOPC/eSM/Chol	0.195 ± 0.009	0.267 ± 0.034	0.088 ± 0.004	2.979 ± 0.587	0.040 ± 0.003
StnI with POPC/PSM	0.157 ± 0.007	0.224 ± 0.020	0.103 ± 0.005	2.273 ± 0.170	0.031 ± 0.002

a The initial anisotropies (*r_i_*) and correlation times (θ_*i*_) of each component together with the limiting anisotropy (*r*_∞_) are indicated. Values were obtained from fitting, and errors from bootstrap analysis.

These same anisotropy decays were then recorded in the presence of DOPC/eSM/Chol LUVs (1:1:1 molar ratio) or POPC/PSM LUVs (4:1 molar ratio). The anisotropy decay of the free label in the presence of lipids was essentially identical (Table 1) to that observed in their absence, indicating that no direct interaction was established between the fluorophore and the vesicles. Energy transfer is a phenomenon that is known to reduce anisotropy.^36−38^ Hence, the fractional labeling of sticholysins was kept low, at 2%, to minimize potential energy transfer between neighboring subunits in the pores. The L:P molar ratio was also high, beyond saturation, so that energy transfer between subunits of different pores was unlikely. At the same time, maximum possible binding of the available toxin was achieved. The observed rapid correlation time did not substantially vary from the previously measured time in any of the subsequent measurements. The slow correlation time was slightly increased when Chol-containing LUVs were used but decreased when LUVs lacking Chol were employed. The limiting anisotropy was larger in both cases, in the range of 0.03–0.04, indicating the restricted mobility of the membrane-bound proteins (Table 1). Correlation times could not be used to calculate molecular sizes this time due to the restrictions imposed by the membrane on the mobility of the proteins.

### Oligomerization in Solution

Previous reports have shown that StnII can oligomerize in solution.^39^ Hence, energy transfer in solution, prior to pore formation on membranes, could be expected to occur, at least to some extent. With this background, and as a first approach, steady-state anisotropy of StnI-ATTO-488 was also measured. Anisotropy is expected to decrease as a consequence of energy transfer.^36−38^ This is consequence of an effective larger displacement of the emission dipole (that of the acceptor molecule) relative to the original absorption dipole (of the molecule acting as a donor), larger than what could be achieved solely by molecular motions. Hence, the steady-state anisotropy of ATTO-488-labeled StnI in solution was measured at increasing degrees of labeling (DoL) (Figure S6). Only at high DoL values did the anisotropy appear to decrease slightly, though not significantly. This effect was more noticeable for membrane-bound toxins (Figure S6).

**Figure 3:**
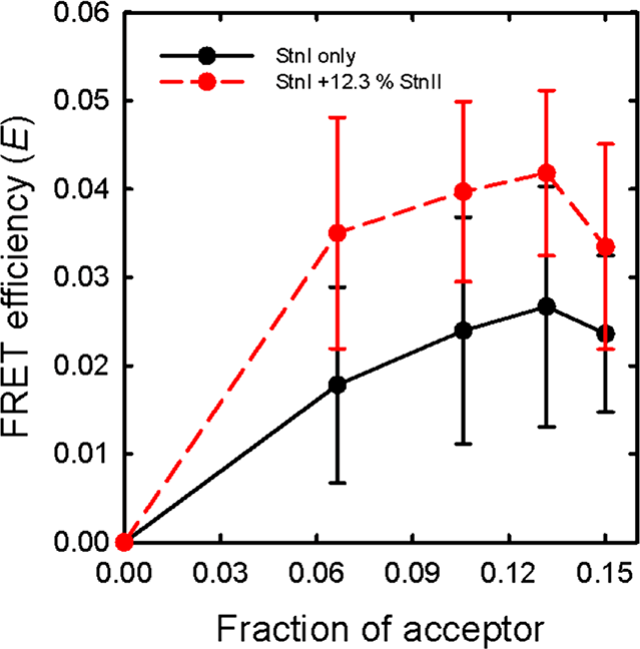
FRET efficiency observed for donor-labeled StnI with WT StnI (solid black trace) or WT StnII (dashed red trace). Initially, both samples contained 11.9% of donor-labeled StnI and 29.1% of the indicated WT variant, with the remainder being unlabeled StnI-T43C. The samples were then titrated with acceptor-labeled StnI (labeled 25.9%) to a final composition of 5% donor, 15% acceptor, and 12.3% WT variant, the remainder being unlabeled StnI-T43C. Values are averages of three replicates ± the standard error of the mean. A version of this figure showing the standard deviation is provided as Figure S7.

Therefore, to improve the resolution of the experiment, a sample of ATTO-488-labeled StnI-T43C (hereafter StnI-488 or donor) was titrated with ATTO-542-labeled StnI-T43C (hereafter StnI-542 or acceptor). The *R*_0_ of this FRET pair was significantly larger than the distance over which the previous approach was effective (only at *r* < 0.8*R*_0_^38^). WT StnI was included to control the exact fractions of donor- and acceptor-labeled populations. The relative fractions of both the donor and the acceptor in the sample varied as a consequence of the titration process, while the total amount of donor (which is responsible for the measured signal) was constant. The measured FRET efficiencies were plotted as a function of the fraction of the acceptor, which was deemed more representative than the fraction of the donor or unlabeled toxin (Figure 3). A small increase in the level of energy transfer was observed as a function of the level of the acceptor in the sample, indicative of the presence of oligomers.

Incidentally, it has been shown that a minimal amount of StnII, just 1%, is capable of greatly enhancing the hemolytic activity of StnI, presumably by facilitating the binding step of the pore formation process.^40^ If that is so, then StnII could also be expected to oligomerize with StnI while in solution. The results of titrating StnI-488 with StnI-542, albeit replacing WT StnI with StnII, showed that the efficiency of energy transfer was more pronouncedly increased than that observed using WT StnI (Figure 3).

### Stoichiometry of StnI Pores from FRET in 1:1:1 DOPC/eSM/Chol LUVs

**Figure 4:**
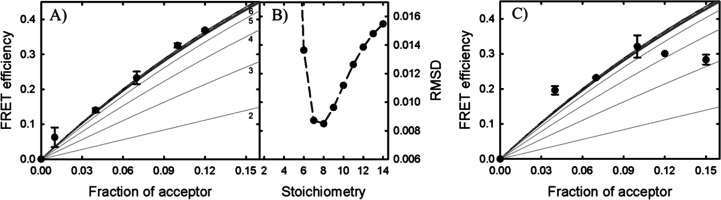
(A) Experimental energy transfer efficiency values (●) obtained using labeled StnI in a combination of 1:1:1 DOPC/eSM/Chol membranes plotted with the theoretical predictions made for stoichiometries from 2 to 10 (11 to 14 removed for the sake of clarity). From bottom to top, predictions for 2, 3, 4, 5, etc., respectively (as indicated in the figure). Predictions from 6 on can barely be distinguished at this scale. With the conditions considered, the maximum FRET efficiency is reached for heptamers, being slowly reduced for higher values (see Figure S9). (B) Root-mean-square deviations (RMSDs) obtained for the experimental values on the left compared to each of the predictions. (C) FRET efficiency values observed for labeled StnI in combination with 4:1 POPC:PSM LUVs. Experimental values do not follow the expected trends.

Energy transfer was also measured directly in sticholysin oligomers assembled on membranes. For that reason, lipid vesicles were added to a 100 nM mixture of StnI to achieve a final L:P molar ratio of 160. The fraction of donor-labeled proteins in the sample was kept constant at 0.05, whereas the fraction of acceptor-labeled monomers was varied between experiments and increased to ≤0.15. FRET efficiency values were obtained from steady-state data using quenched donor emission as measured from the deconvoluted total sample emission (Figure S8). These values were plotted as a function of the acceptor content in the sample and compared with the theoretical predictions (Figure 4a). To properly evaluate which of the theoretical traces better described the experimental data, the RMSD was calculated for the experimental values relative to each of the theoretical traces, using the error of each experimental value for weighting the calculation (Figure 4b). The signal of StnI pores on 1:1:1 DOPC/eSM/Chol membranes best agreed with that of octameric ensembles. However, experimental resolution and model limitations did not permit us to rule out the formation of oligomers equal to or larger than heptamers, based solely on this result.

### Is the Stoichiometry of StnI Pores from FRET in 4:1 POPC/PSM Membranes Different?

The experiment and subsequent analysis were repeated with vesicles without Chol, to determine if the presence of Chol had a substantial effect on oligomerization. This time, the L:P molar ratio was increased to 320, in spite of increased lipid-induced light scattering, to ensure complete binding due to the comparatively lower affinity of StnI for membranes lacking Chol.^41^ Surprisingly, the results showed that the signal from StnI did not follow the trend expected from any of the theoretical predictions (Figure 4c), indicating that the assumptions of the model are not valid for this situation.

### A 12% StnII in the StnI Sample Does Not Affect the Stoichiometry

**Figure 5:**
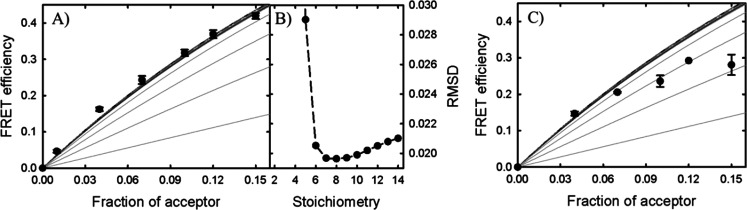
(A) Experimental energy transfer efficiency values (●) obtained using labeled StnI with 12.3% StnII in a combination of 1:1:1 DOPC/eSM/Chol membranes plotted with the theoretical predictions as in Figure 4. (B) RMSDs for each of the predictions. In this case, the difference observed between the stoichiometries is reduced compared with the previous result in the absence of StnII. (C) FRET efficiency values observed for labeled StnI in the presence of 12.3% StnII in combination with 4:1 POPC/PSM LUVs. Again, the experimental values do not follow the expected trends.

The experiment was performed again for each lipid composition. This time, however, the sample included 12.3% StnII, to ascertain the stoichiometry of the pores formed when using a mixture of both proteins. The results showed that the presence of StnII did not change the trends observed in its absence (Figure 5a,c). The distribution of RMSD values of the experiment performed in the presence of Chol (Figure 5b) closely resembled that observed in the absence of StnII, indicating that the stoichiometry is conserved in Chol-containing membranes. The unexpected experimental trend that we had observed for membranes without Chol was also maintained, again showing that the theoretical assumptions regarding the process of pore formation do not adequately describe the process that takes place in the absence of Chol (Figure 5c).

### Sticholysin Pores Are Not Remodeled Once Formed

The results from studies using electrophysiological measurements suggested that sticholysin pores might be unstable, based on the noise level of the conductance measured compared with those observed for β-pore-forming toxins.^42^ One possibility is that this noisiness was simply caused by thermal oscillations of the system, because the lumen of the pore would be lined by the N-terminal α-helices of sticholysins and lipids, with few direct interactions between the components other than van der Waals forces. However, the possibility that, once formed, pores would be under continuous remodeling, with monomers moving from one complex to another, has also been proposed.

**Figure 6:**
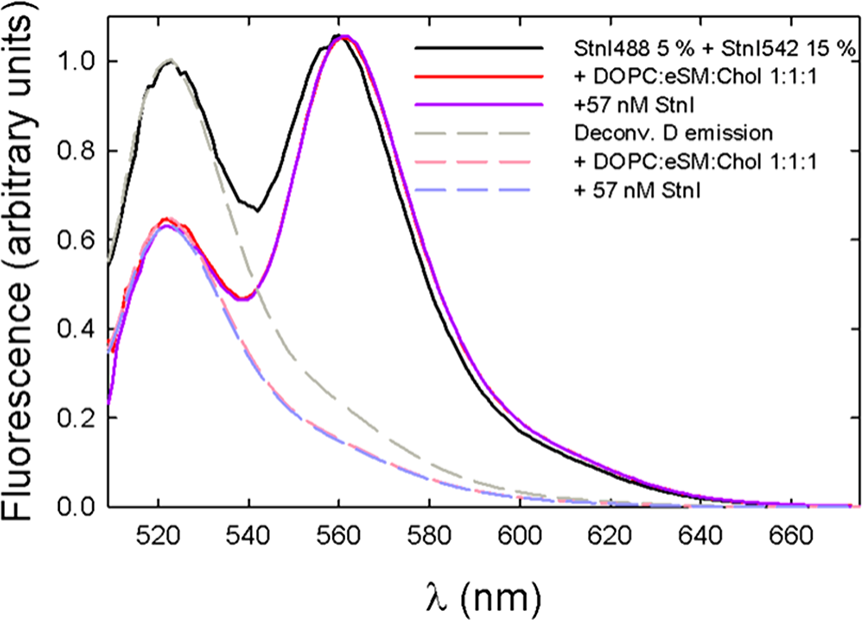
Emission spectra of StnI with 5% donor labeling and 15% acceptor labeling in solution (solid black line), with 1:1:1 DOPC/eSM/Chol membranes (solid red line), and after addition of extra 57 nM unlabeled StnI (solid purple line). Deconvoluted donor emission is also shown as the corresponding dashed line. The later addition of StnI did not affect the FRET efficiency, indicating that pores are not remodeled.

**Figure 7:**
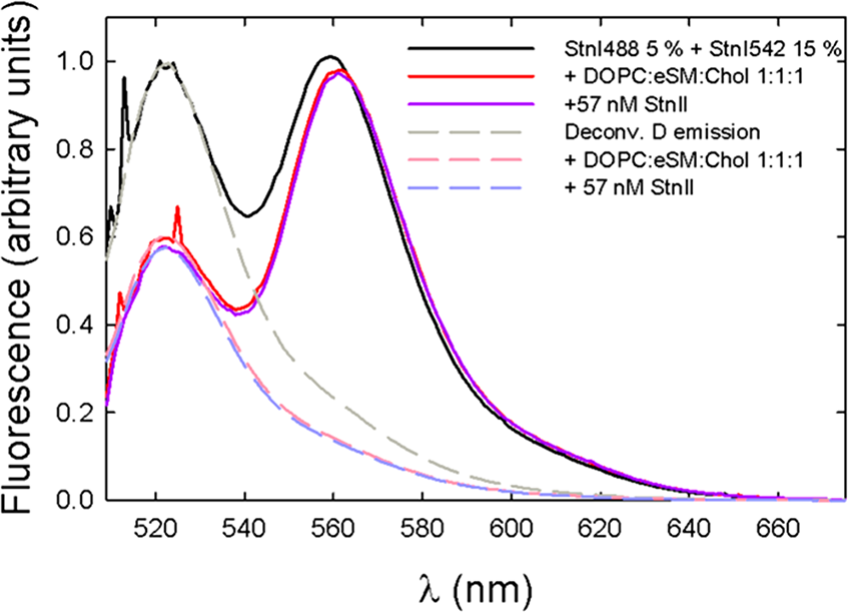
Emission spectra of StnI with 5% donor labeling and 15% acceptor labeling in solution (solid black line), with 1:1:1 DOPC/eSM/Chol membranes (solid red line), and after addition of extra 57 nM unlabeled StnII (solid purple line). Deconvoluted donor emission is shown as the corresponding dashed line. As for StnI, the addition of StnII did not affect FRET efficiency, indicating stable pores.

To test this idea, StnI or StnII, to a final increment of 57 nM, was added to 100 nM StnI preincubated with lipids, already including 5% donor and 15% acceptor proteins. The L:P molar ratio was initially set to 240. That way, the final L:P molar ratio after the addition of the extra protein was 160, comparable to the results of the assays described above. In neither case did the observed FRET efficiency vary after the addition of extra unlabeled protein, regardless of whether it was StnI or StnII (Figure 6 and 7, respectively). This suggested that once equilibrium was reached, previously formed pores were not disturbed by newly added toxin molecules and maintained the same monomers that oligomerized in the first place, resulting in a constant FRET signal.

## Discussion

In this study, several aspects of the process of pore formation by sticholysins were investigated using the single-cysteine StnI mutant T43C, labeled with ATTO-488 and ATTO-542. Using time-resolved measurements, the hydrodynamic size of StnI was measured as 29.0 ± 1.3 Å, which is in good agreement with the dimensions of a sticholysin monomer according to structural determination techniques.^27,28^ The anisotropy decay of the soluble protein reveals a correlation time that agrees with the molecular size of the protein but also shows that some oligomers are present, as indicated by the fact that the limiting anisotropy was slightly larger than 0 (Figure 2 and Table 1). These results agree with the previous observations on StnII, showing that it can oligomerize up to tetrameric ensembles in solution while mainly maintaining a monomeric nature.^39^

Delving further into that line of research, titration experiments were used to corroborate the presence of oligomers in solution. Using solely StnI, the presence of oligomers was detected as a slight increase in FRET efficiency. When StnII was included, a further increase in the efficiency of energy transfer was observed (Figure 3). This result supports the previously observed behavior indicating that StnII promotes the binding of StnI to the membrane, presumably by binding to it while still in solution.^40^ This result suggests not only that the number of oligomers in solution is larger but also that there are higher-order oligomers, such as trimers and tetramers (given that StnII was not labeled, it could increase FRET efficiency only if trimers and/or tetramers occur).

The FRET approach was then taken one step further to calculate the stoichiometry of sticholysin pores in lipid model vesicles. The signal from labeled-StnI in 1:1:1 DOPC/eSM/Chol membranes was best described by the theoretical prediction that assumed an octameric complex. However, due to physical constraints, namely, the *R*_0_ of the FRET pair and the distance between labels on neighboring subunits, the prediction of the model became increasingly similar as the oligomerization number was increased (Figure S9). These experimental results rule out the existence of a detectable number of oligomers of five or fewer subunits, but simultaneously, any larger stoichiometry should not be entirely discarded, even if the prediction obtained under the assumptions of octamers is the one that best agrees with the experiment (Figure 4a). Using a strategy similar to ours but based on spin labeling and electron paramagnetic resonance spectroscopy, it has been recently concluded that StnI in a membrane would exhibit an oligomeric architecture with heterogeneous stoichiometry of predominantly eight or nine protomers, which agrees with the available structural models.^33,43^ In fact, the results presented here are compatible with the possibility that several other stoichiometries occurred at once, yielding the same signal as if all were octamers. It is also important to remark that our approach observes oligomeric structures in equilibrium, without contemplating the trajectory on the membrane followed by the different oligomeric structures during pore formation and/or remodeling before reaching their stable final assembly.

**Figure 8:**
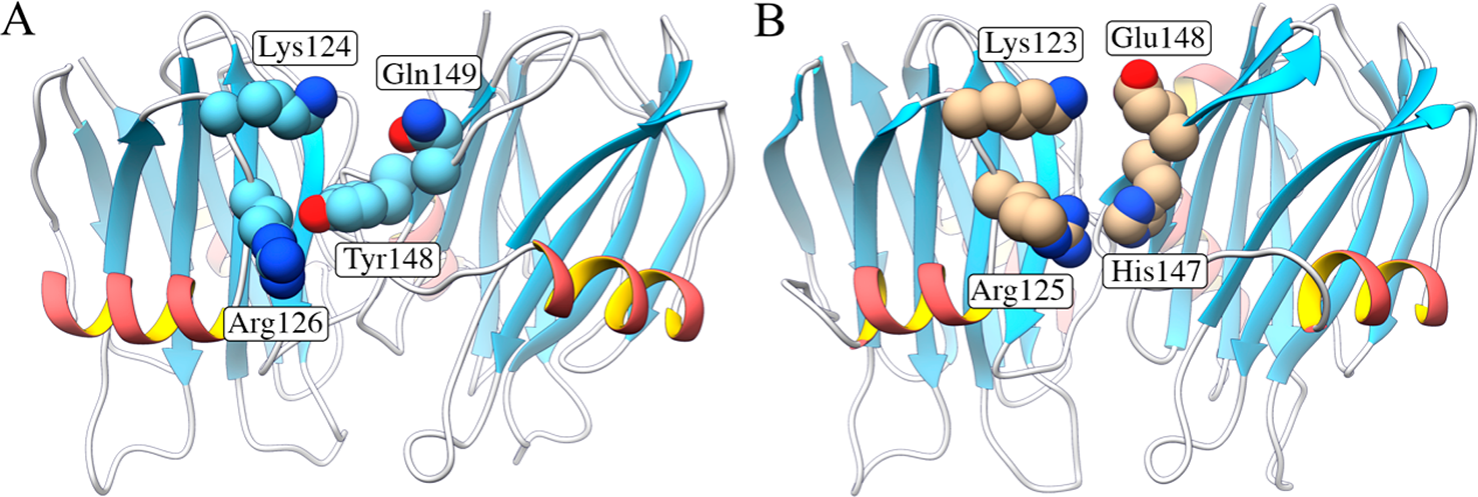
Model homodimers of (A) StnI and (B) StnII. The residues shown are the same in both cases for the monomer on the left, Lys124 and Arg126 of StnI (Lys123 and Arg125 of StnII). For the monomer on the right, the amino acids differ. For StnI, they are Tyr148 and Gln149, whereas for StnII, they are His147 and Glu148. The orientation of the residues is not necessarily that found upon oligomerization, because the structures were obtained in solution (A) and by crystallization (B). The Arg residue could interact with either of the aromatic residues, by hydrogen bonding or by a cation−π interaction. The latter can, in fact, be observed in the dimer structure published for FraC (PDB entry 4TSL(32)) between its equivalent amino acids. The Lys residue probably forms a hydrogen bond with Gln149 and a salt bridge with Glu148. This figure was made using the three-dimensional structures of StnI (PDB entry 2KS4) and StnII (PDB entry 1GWY), superimposing them with the octameric pore of FraC (PDB entry 4TSY), which has been shown to be compatible with the stoichiometry of sticholysins.

Interestingly, the inclusion of 12.3% StnII did not affect the distribution of the RMSD values observed for StnI alone, indicating that StnII did not affect the overall stoichiometry of the pores at equilibrium. It could be argued that StnII did not oligomerize with StnI. However, this would contradict the observed ability of StnII to enhance StnI binding^40^ and imply that the observed signal should shift upward because the partial fraction of labeling would be larger in the StnI-only complexes than accounted for in the calculations, which assume that StnII is part of the total toxin population. This effect was not observed. In fact, the presence of oligomeric structures containing both StnI and StnII has been detected before, in the presence of vesicles with a composition identical to the ones used now, employing cross-linking experiments.^40^ This is the first time, however, that it has been shown that the stoichiometry is conserved when both proteins are mixed. This fact also suggests that the essential residues responsible for protein–protein interactions should be conserved between both sticholysin isoforms. At this point, it is interesting to remember that oligomerization is promoted by the presence of StnII and that two of the only 12 residues that differ between StnI and StnII are located very close to or at the protein–protein interfaces. These two residues are Tyr148 and Gln149 in StnI and His147 and Glu148 in StnII. Overall, the properties of these amino acids are conserved in both residue pairs. On this basis, we can predict the nature of the complementary residues located on the other side of the protein. Those should be capable of both hydrogen bonding and perhaps also establishing salt bridges, and one of them would have to be cationic so that it could establish a cation−π interaction with the aromatic rings observed. Inspection of the three-dimensional structures reveals that there is, in fact, such a pair of residues, appropriately standing out from the protein. These are Arg126 and Lys124 in StnI and Arg125 and Lys123 in StnII. So far, the difference in activity between StnI and StnII has been attributed, to a very large degree, to the different strength of attachment of the N-terminal α-helix to the β-sandwich, as well as to its different hydropathy profile.^35^ However, it is also possible that a stronger monomer–monomer interaction, which is feasible only with the residues of StnII (a possible salt bridge between Glu148 and Lys123/Lys124), could intervene, shifting the equilibrium in solution to the multimeric forms, which could in turn favor membrane binding and, certainly, oligomerization (Figure 8).

The results obtained when using membranes that lack Chol are, at first glance, surprising. In all cases, the experimental values do not follow the predictions. In addition, their trend does not agree with what would be expected if the pores clustered, which would be higher than expected *E* values, which would be the consequence of an increased probability of energy transfer between different oligomers due to their higher-than-expected proximity. The fact that the first points, corresponding to the lower acceptor fractions, are closer to the estimations and then cease to rise accordingly, at larger fraction values, could indicate that the binding unit for membranes without Chol would be sticholysin dimers/oligomers. At low acceptor levels in the sample, the expected *E* is less dependent on the presence of acceptors at subunit *i* + 1 (from a donor placed at *i*). However, the more the acceptor fraction increases, the greater the relevance is of the probability of finding acceptors, simultaneously, at positions *i* + 1 and *i* – 1. Because donor- and acceptor-labeled proteins are mixed right before the measurements are performed, and in the total final volume, complete shuffling of the variants in the oligomers might not occur quickly enough. Consequently, oligomers in the sample would rarely have donor–acceptor pairs, yielding the observed effect. This is compatible with previous results indicating that, in fact, dimers are required for membrane binding in the absence of Chol.^33,44^ It is, therefore, possible that Chol’s effect on SM’s headgroup orientation^45^ aids in SM recognition, permitting the dominant form in solution, monomers, to directly bind those membranes. We confirmed that, in all cases, FRET was a consequence of specific membrane binding and SM recognition by incubation of a labeled sample with POPC vesicles, which did not affect the emission of the sample (Figure S10).

Finally, we have shown that sticholysin pores are stable once in equilibrium and do not undergo any kind of subunit exchange when new monomers are added to the sample. This certainly does not happen on the time scale used (2–5 min), which is much greater (seconds) than that used in the electrophysiology experiments that prompted the question.

## Conclusions

In this work, we have presented evidence disproving that tetramers are the oligomerization assembly of StnI at equilibrium. The inclusion of 12.3% StnII showed no significant effect on the stoichiometry-dependent energy transfer efficiency, indicating that the stoichiometry is conserved regardless of the composition of the pores. Both of these results would support the X-ray structure obtained for FraC, suggesting that the assembly is probably the same for all known actinoporins.

## Supporting Information Available

Structural and functional characterization of the StnI-T43C mutant, selection of the mutated residue and reach of the label, absorption spectra of the mutant StnI-T43C before and after labeling, overlap of the anisotropy and intensity decays of the labeled toxin in solution and on membranes, schematic representation of the model used to calculate FRET efficiencies on membranes, and dependence of the steady-state anisotropy on the degree of labeling (in solution and on membranes, example of fluorescence signal deconvolution, dependence of predicted FRET on the stoichiometry for 15% acceptor in the sample, and negative control for the activity of the labeled mutants) (PDF)

## Accession Codes

StnI, UniProtKB P81662; StnII, UniProtKB P07845.
