# Supplementary material for: Oligomerization of Sticholysins from Förster Resonance Energy Transfer
Source: Biochemistry. 2021 Jan 14;60(4):314–23. doi: 10.1021/acs.biochem.0c00840 (PMC8023573; doi:10.1021/acs.biochem.0c00840)
Supplement: Supplementary file 1 — bi0c00840_si_001.pdf [file bi0c00840_si_001.pdf]

Supplementary Information File for

Oligomerization of sticholysins from Förster resonance energy transfer

Juan Palacios-Ortega,*[1,2] Esperanza Rivera-de-Torre,[1] Sara García-Linares,[1] José G. Gavilanes,[1] Álvaro Martínez-del-Pozo,[1] J. Peter Slotte[2]

[1] Depto. de Bioquímica y Biología Molecular, Universidad Complutense, Madrid, Spain
[2] Biochemistry, Faculty of Science and Engineering, Åbo Akademi University, Turku, Finland

Corresponding author: Juan Palacios-Ortega
Email: juan.palaciosb1a@gmail.com (ORCID 0000-0002-4629-0221)

This file includes:

Figures S1 to S10

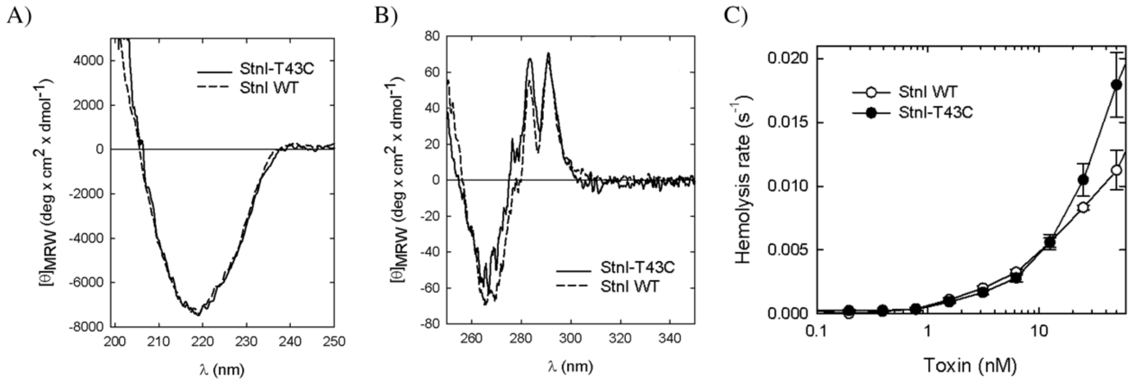

Figure S1. Structural and functional characterization of the StnI-T43C mutant. A) Far-UV circular dichroism of the mutant StnI-T43C (solid line), compared with that of the WT variant of StnI (dashed line). B) Near-UV circular dichroism of StnI-T43C. compared to that of StnI-WT (lines as in A). C) Hemolysis rates displayed by preparations of erythrocytes when exposed to different concentrations of StnI WT (open circles) or StnI-T43C (solid circles).

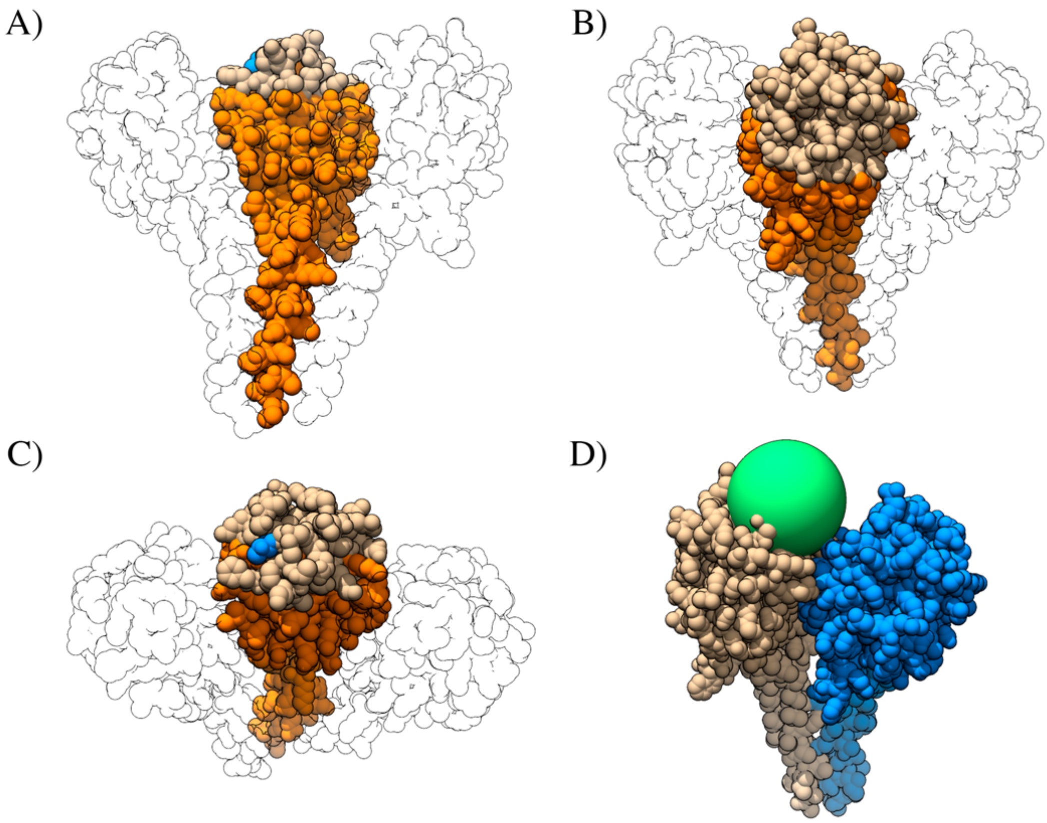

Figure S2. A) Space-filling representation of three StnI monomers (PDB ID: 2KS4) fitted to the structure of the octameric pore of FraC (PDB ID: 4TSY), as viewed from the lumen of the pore. The residues that were discarded due to their potential implication in the functionality of StnI, i.e. membrane binding, structural transition of the N-terminal α-helix, and monomer-monomer interactions, are shown in orange. Those that were supposedly not involved in the aforementioned functions are shown in tan. Residue T43 is highlighted in blue. B) Back view of the same structure depicted in A). C) Top view of the structure depicted in A). D) Two monomers of the structure in A) have been isolated and, in one of them, a sphere of 12 Å radius, corresponding to the approximate maximum length of the probes used, has been centered at the oxygen atom of T43. It can be seen that the probe would not interfere in the interaction with the neighboring monomer.

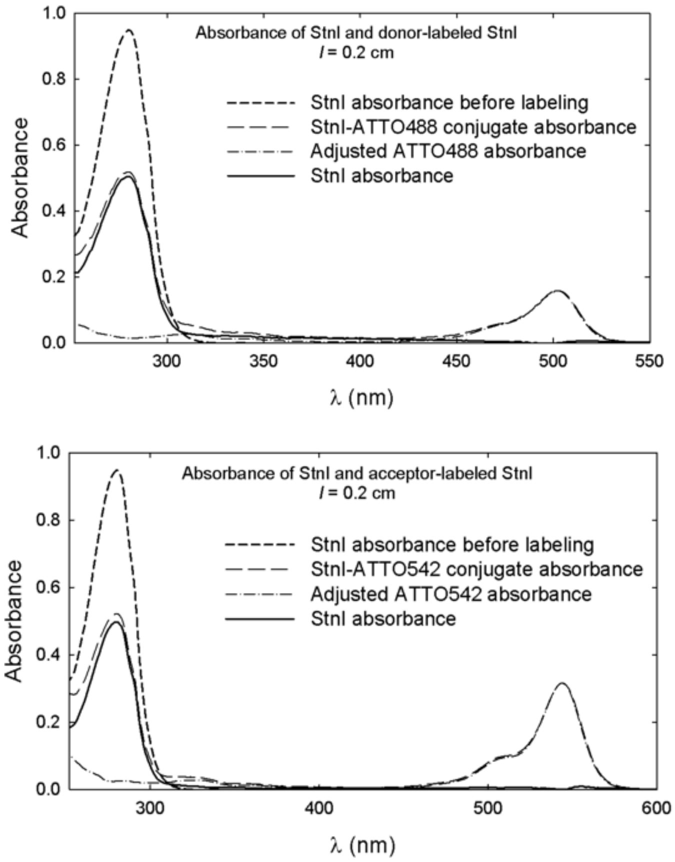

Figure S3. Absorption spectra of StnI-T43C before and after labeling with ATTO probes. Top: absorbance of StnI-T43C before and after labeling with ATTO-488. The absorption of the protein corrected for probe contributions is also shown. Bottom: absorbance of StnI-T43C before and after labeling with ATTO-542. The absorption of the protein corrected for probe contributions is also shown. Notice the different scale on the *x*-axis between both graphs.

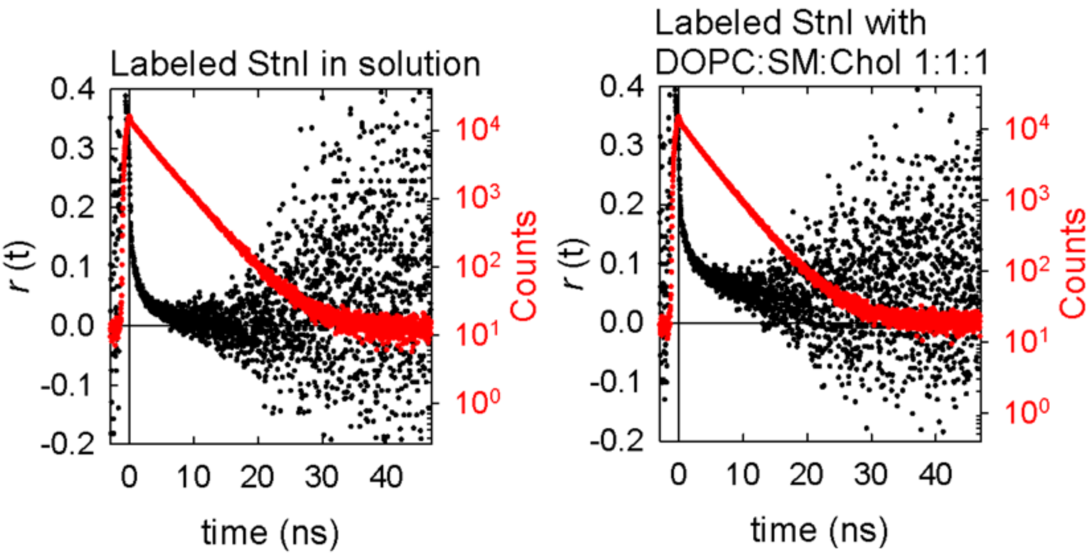

Figure S4. Anisotropy (black) and intensity (red) decays of ATTO-488 labeled StnI-T43C in solution (left) and bound to DOPC:SM:Chol (1:1:1 molar ratio) LUVs (right). In both cases, the anisotropy decay is over before the intensity decay, indicating that the order parameter κ^2, required to calculate the Förster distance (R_0) can be set to 2/3, according to the dynamic isotropic limit. Notice the logarithmic scale for the intensity decay.

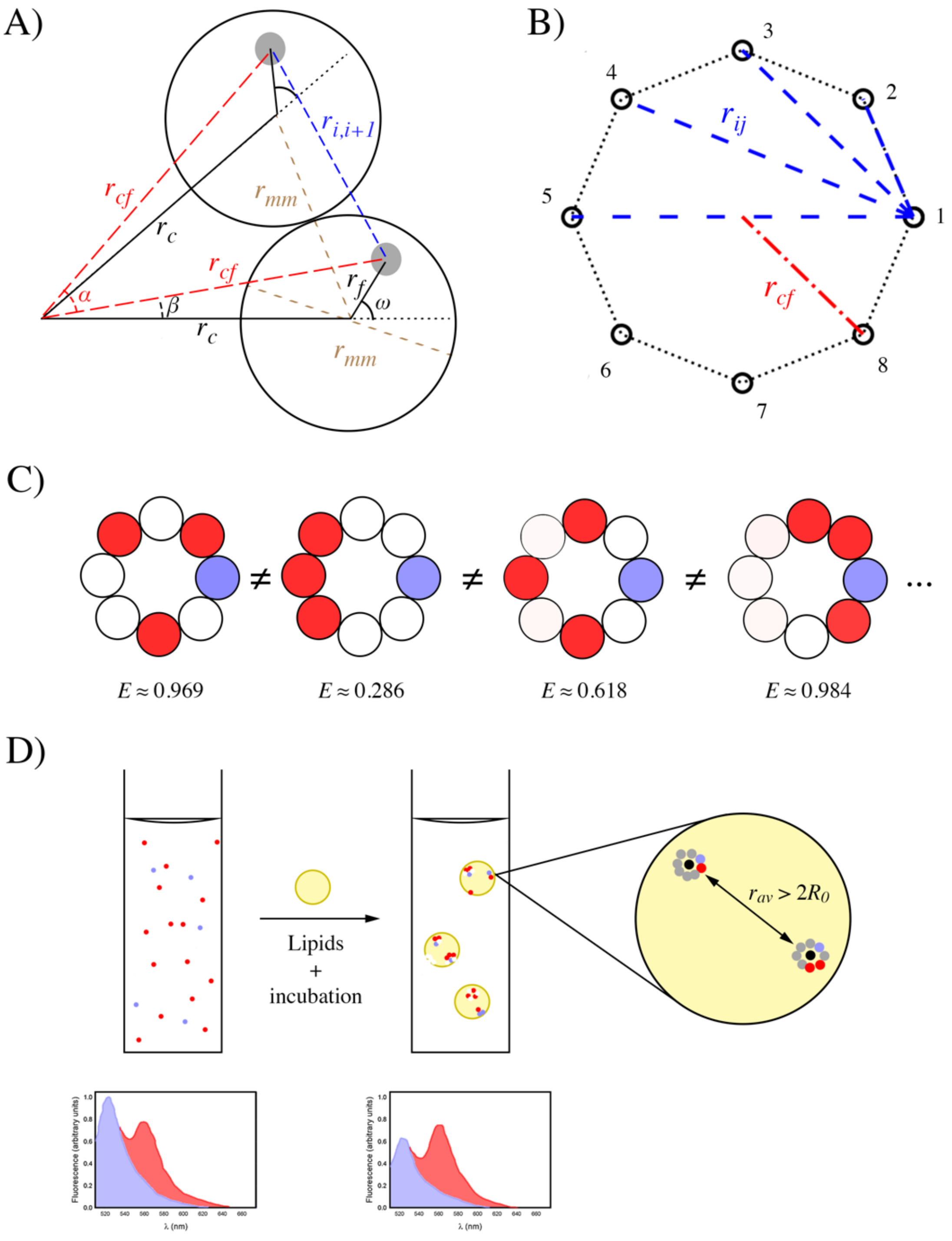

Figure S5. A) Representation of the geometrical arrangement of the two labeled proteins in a given n-mer. Only two subunits are shown. Fluorophores are depicted in grey. Parameters shown are those in eqs. 5 to 7. The value of r_{mm}, the distance between the centers of two adjacent subunits, is equal to the diameter of one subunit. This cannot be applied to the position of the fluorophores, since they are not placed at the center of the protein. Instead, they have an offset of r_f, at an angle ω, For that reason, $r_{mm} \neq r_{i,i+1}$. This is solved by using eq. 6., which enables to calculate r_{cf}, enabling to calculate the correct values for $r_{i,j}$. The angle α has a value of 2π/N radians, where N is the number of subunits of the oligomer. The angle β represents the offset of r_{cf} relative to r_c, and is a function of r_c, r_f, and ω. It is because of this offset that r_{mm} and $r_{i,i+1}$ are not parallel. This scheme is valid for any n-mer. B) Representation of an exemplary oligomer, an octamer. Circles represent fluorophores in oligomerizing proteins (omitted). The

parameter $r_{i,j}$ is represented by blue dashed lines. Some are omitted for clarity. C) Following the example given in the main text of eight subunits, with the N_D = 1 (in purple), N_A = 3 (in red), and N_U = 4 (in white), we have that those can assemble into 35 possible arrangements (discarding those that only differ by circular symmetries). Four of those arrangements are depicted in the figure. In our model, we assume that the probability of those subunits (N_D = 1, N_A = 3, and N_U = 4) assembling into any of the 35 possible arrangements is the same. However, the FRET that a donor experiences from each arrangement is different (see figure), hence the need to average them. Then, that average need to be weighted, since the probability of an assembly as those considered (regardless of the arrangement) is different to that of those with a composition, say, N_D = 1, N_A = 1, and N_U = 6. Those probabilities depend on the fractional population of donor- and acceptor-labeled proteins in the sample. D) Scheme of the experimental design. Non-labeled toxins, together with donor- and acceptor-labeled toxins are placed in a cuvette, and the emission is registered (colors as in C). After that, lipids are added. The sample is left for a few minutes to ensure that binding is complete. After that, emission is registered again, revealing FRET from a diminished donor emission. The L/P ratio is such that the average distance, r_{av}, between oligomers is, at least, larger than $2R_0$.

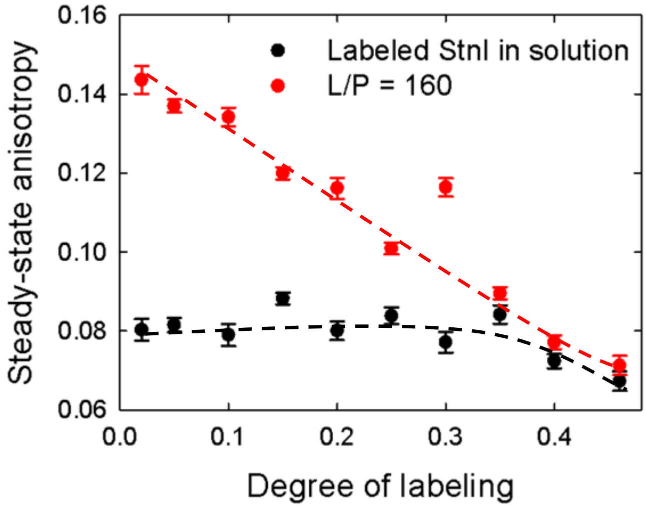

Figure S6. Steady-state anisotropy was dependent on the degree of labeling of StnI with ATTO-488, in solution (solid black dots, the dashed line is a guide to the eye) and, especially, when bound to membranes (solid red dots, the dashed line is a guide to the eye).

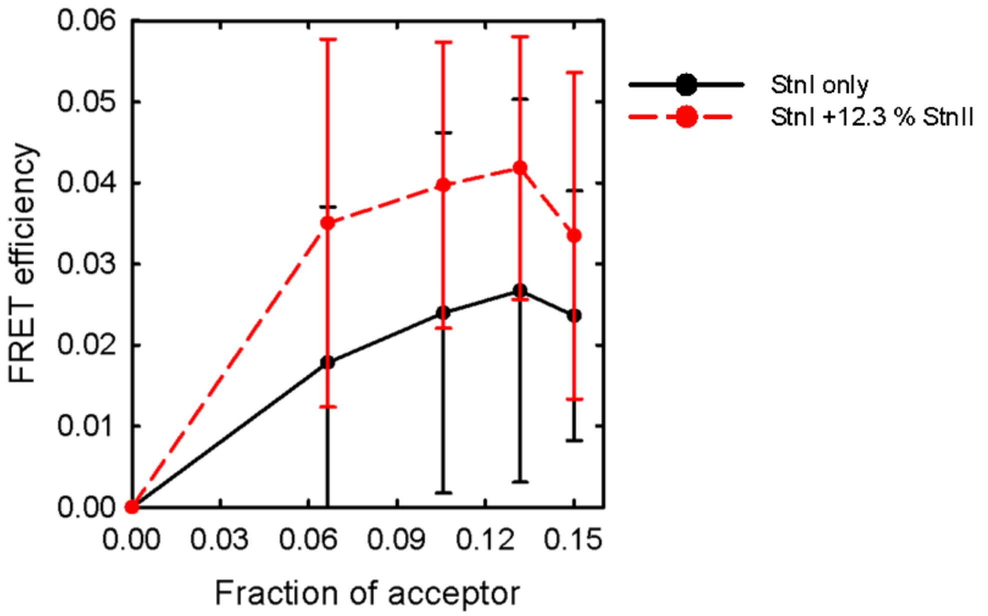

Figure S7. FRET efficiency observed for donor-labeled StnI with WT StnI (solid black trace) or with WT StnII (dashed red trace). Initially, both samples contained 11.9% of donor-labeled StnI, and 29.1% of the indicated WT variant, with the remainder being unlabeled StnI-T43C. The samples were then titrated with acceptor-labeled StnI (labeled 25.9%) to a final composition of 5% donor, 15% acceptor, and 12.3% of the WT variant used, the remainder being unlabeled StnI-T43C. Values are average of n = 3 (SD).

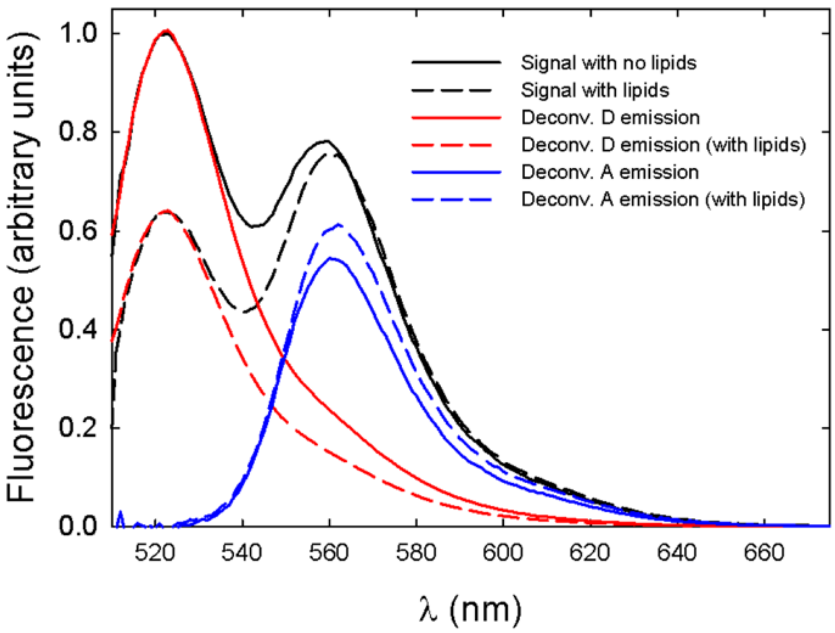

Figure S8. Example of signal deconvolution. The represented spectra have all already been corrected for arbitrary signal contributions, such as Raman peak and scattering from lipids. The deconvoluted emissions of the donor (red) were used to calculate the FRET efficiency for each sample.

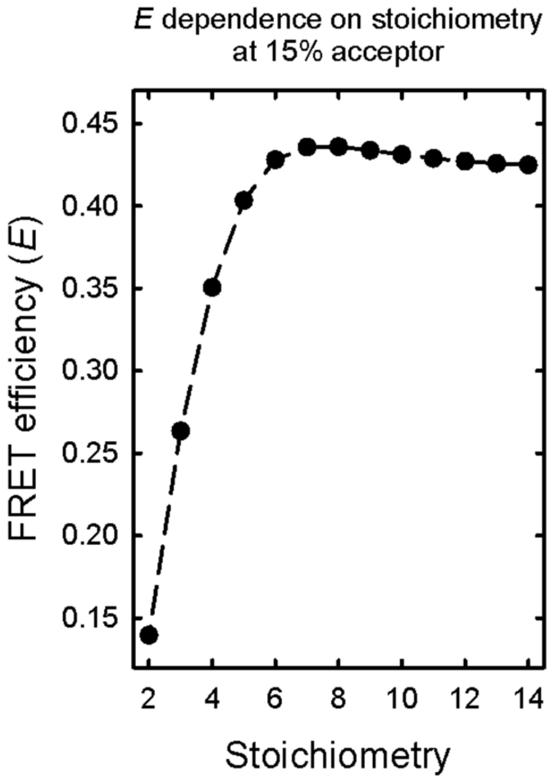

Figure S9. Predicted change of the FRET efficiency at 15% acceptor content in the sample as a function of complex stoichiometry, given the physical restrictions imposed by the size of sticholysins, the labeling position, and the photophysical characteristics of the labels used. The differences in the predictions were also reduced as the acceptor content was increased.

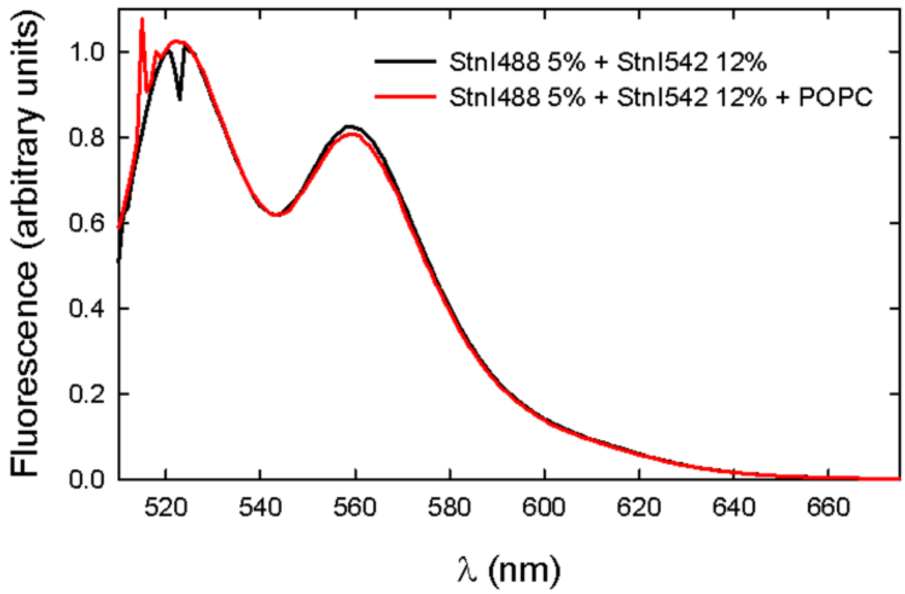

Figure S10. Fluorescence emission of a sample containing 5% of StnI488 and 12% of StnI542 before (black line) and after (red line) incubation with POPC vesicles to a final L/P molar ratio of 320. Emission did not change after the inclusion of lipids, revealing absence of membrane-binding and associated FRET.
